# Hierarchically Multifunctional Polyimide Composite Films with Strongly Enhanced Thermal Conductivity

**DOI:** 10.1007/s40820-021-00767-4

**Published:** 2021-12-10

**Authors:** Yongqiang Guo, Hua Qiu, Kunpeng Ruan, Yali Zhang, Junwei Gu

**Affiliations:** Shaanxi Key Laboratory of Macromolecular Science and Technology, School of Chemistry and Chemical Engineering, Northwestern Polytechnical University, Xi’an, 710072 Shaanxi People’s Republic of China

**Keywords:** EMI shielding, Flexible films, Polyimide, Thermal conductivity

## Abstract

The development of lightweight and integration for electronics requires flexible films with high thermal conductivity and electromagnetic interference (EMI) shielding to overcome heat accumulation and electromagnetic radiation pollution. Herein, the hierarchical design and assembly strategy was adopted to fabricate hierarchically multifunctional polyimide composite films, with graphene oxide/expanded graphite (GO/EG) as the top thermally conductive and EMI shielding layer, Fe_3_O_4_/polyimide (Fe_3_O_4_/PI) as the middle EMI shielding enhancement layer and electrospun PI fibers as the substrate layer for mechanical improvement. PI composite films with 61.0 wt% of GO/EG and 23.8 wt% of Fe_3_O_4_/PI exhibits high in-plane thermal conductivity coefficient (95.40 W (m K)^−1^), excellent EMI shielding effectiveness (34.0 dB), good tensile strength (93.6 MPa) and fast electric-heating response (5 s). The test in the central processing unit verifies PI composite films present broad application prospects in electronics fields.

**Supplementary Information:**

The online version contains supplementary material available at 10.1007/s40820-021-00767-4.

## Highlights

- Hierarchically multifunctional polyimide composite films were fabricated by hierarchical design and assembly strategy.
- Polyimide composite films have three functional layers and integrates high thermal conductivity (95.40 W (m K)^−1^), excellent EMI shielding (34.0 dB) and good tensile strength (93.6 MPa).
- Polyimide composite films present broad application prospects in electronics fields according to the test results in the central processing unit.

**Supplementary Information:**

The online version contains supplementary material available at 10.1007/s40820-021-00767-4.

## Graphical abstract

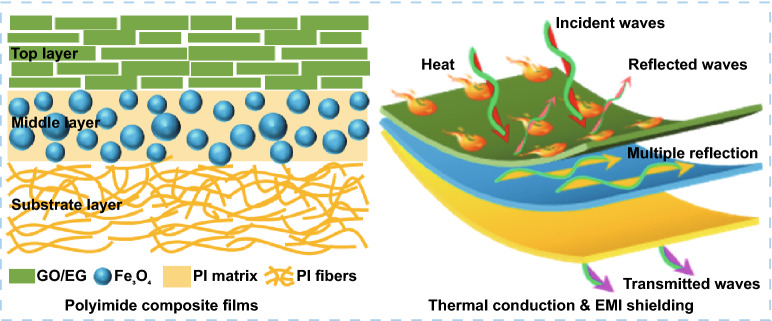

## Introduction

The development of lightness and high integration for electronics inevitably causes serious heat accumulation (> 5 W cm^−2^) [1] and electromagnetic interference (EMI), which trigger increasing demand for effective heat dissipation and EMI shielding at the multifunctional materials level [2–4]. In this context, polymer-based composite films integrating excellent thermal conduction and EMI shielding are expected to meet the requirement of electronics manufacturing due to their advantages of lightweight, easy processing and good designability [5–7].

Many single or hybrid fillers with different functions (thermal, electrical, magnetic [8–10]) have been added into polymer matrix by molding press, filtration, blade coating, etc*. *[11–13], to fabricate polymer-based composite films with enhanced thermal conduction and EMI shielding performances [14–18]. Wei et al*.* prepared multi-walled carbon nanotube/water-based polyurethane (MWCNT/WPU) composite films by *in situ* polymerization and blade coating method. In the presence of 48.1 wt% MWCNT, the in-plane thermal conductivity coefficient (*λ*_∥_) and EMI shielding effectiveness (SE) of MWCNT/WPU composite films with thickness of 80 μm reached 5.31 W (m K)^−1^ and 65.3 dB, respectively [19]. Fu et al*.* filtrated the mixture of graphene oxide (GO) and cellulose nanofibers (CNF) and reduced it to prepare rGO/CNF composites films. The *λ*_∥_ and EMI SE of the rGO/CNF composite films with 50 wt% rGO were 7.30 W (m K)^−1^ and 26.2 dB, respectively [20]. However, directly mixing the fillers and polymer has obvious disadvantages, which is difficult to form efficient fillers thermal conduction pathways and networks because the fillers are easily separated or coated by the polymer matrix [21]. Moreover, high interfacial thermal resistance between the fillers and polymer matrix results in low thermal conductivity for the prepared composites. In addition, introduction of a large number of thermally conductive fillers is likely to cause poor mechanical and processing properties and higher cost and density, and the enhancement in thermal conductivity is relatively limited [22].

To solve the abovementioned problems, researchers directly construct fillers thermal conduction pathways to prepare fillers films, and then combining them with polymer to fabricate hierarchical composite films [23]. Wang et al*.* attached graphite nanosheets (GNPs) to nylon gauze by filtration method, and then prepared two-layer structured GNPs/nylon composite films by combing hot pressing. When the amount of GNPs was 11.8 wt%, the *λ*_∥_ and EMI SE of the GNPs/nylon composite film reached 15.8 W (m K)^−1^ and 58.1 dB, which were 63 times and 157 times that of pure nylon, respectively. However, its tensile strength was only 32.0 Mpa [24]. Liu et al*.* prepared multi-hierarchical structured GNPs/CNF composite films by alternate filtration method. The *λ*_∥_, EMI SE and tensile strength of the GNPs/CNF composite film with 25 wt% GNPs were 33.55 W (m K) ^−1^, 27.4 dB and 122.4 MPa, respectively [25]. However, these hierarchical composite films are easy to peel off and have poor durability due to the weak force among the fillers [26].

Electrospun polymer fibers have diameters ranging from sub-nanometers to micrometers, the high orientation of polymer molecular chains gives the fibers good mechanical properties and excellent flexibility [27]. Hou et al*.* prepared electrospun polyimide (PI) fibers to reinforce epoxy resin (EP). The tensile strength of PI/EP composites with 30 wt.% PI fibers was as high as 227.3 MPa, which was 511% higher than that of pure EP (37.2 MPa). Therefore, the introduction of polymer fibers into composite films is expected to improve their mechanical properties further [27].

Expanded graphite (EG) possesses excellent thermal conductivity and outstanding EMI shielding performances. However, its brittle nature, difficult assembly and poor mechanical properties limit the preparation and wide application of EG film [28, 29]. To our knowledge, GO has high specific area and could form π-π interaction with EG [30]. The stable dispersion ability of GO in water can promote the dispersion of EG and reduce its agglomeration [31]. Flexible GO films have been proven to be dense and can sustain repeated bending, which could endow EG film with good mechanical properties and flexibility via forming hetero-sheet bonding and introducing foldable and stretchable wrinkles. Besides, excellent thermal conductivity and outstanding EMI shielding of EG film could also be maintained. However, high electrical conductivity of EG can cause excessive reflectivity in electromagnetic waves, resulting in secondary radiation to the environment [8, 32]. It’s necessary to increase the loss of electromagnetic waves to reduce secondary radiation pollution [33], such as combining magnetic fillers with EG [34].

Herein, a hierarchical design and assembly strategy was adopted to fabricate hierarchically multifunctional PI composite films with high thermal conductivity, excellent EMI shielding and good mechanical properties. The top layer of PI composite films was GO/EG, fabricated by the filtration and molding press method, whose main function was thermal conduction and EMI shielding. The introducing of GO into EG films solved the brittle nature of EG films, leading to the balance of mechanical properties, thermal conductivity and EMI shielding performances by optimizing the ratio of GO and EG. The substrate layer of PI composite films was electrospun PI fibers to enhance the mechanical properties. The top and substrate layer were combined with Fe_3_O_4_/PI prepared by in situ polymerization and blade coating, improving the EMI shielding performance. The effects of the amount of each layer on the thermal conductivity, EMI SE, mechanical properties and the electric-heating response of the PI composite films were analyzed. In addition, the potential application of the films in thermal management was also demonstrated by experimental test.

## Experimental Methods

### Materials

Graphite (> 100 mesh, 99.95%) and ammonium persulphate ((NH_4_)_2_S_2_O_8_, 99%) were both purchased from Shanghai Aladdin Bio-Chem Technology Co., Ltd., China. Ferroferric oxide (Fe_3_O_4_, 99.5%) was provided by Shanghai Macklin Biochemical Co., Ltd., China. Pyromellitic dianhydride (PMDA, 99%) and 4, 4′-oxydianiline (ODA, 99%) were both supplied by Beijing J&K Scientific Co., Ltd., China. Dimethylacetamide (DMAc, 99%) was purchased from Guangdong Guanghua Technique Co., Ltd., China. Concentrated sulfuric acid (H_2_SO_4_, 95–98%), potassium permanganate (KMnO_4_) and hydrogen peroxide (H_2_O_2_, 30%) were all supplied by Beijing Chemical Works, China. All materials were used without further purification.

### Preparation of GO/EG Films

GO was prepared by the Hummers method [28]. Graphite was treated by the (NH_4_)_2_S_2_O_8_ to prepare EG [35]. 2 g of graphite and 80 mL of H_2_SO_4_ were mixed and stirred at 35 °C, and then 10 g of (NH_4_)_2_S_2_O_8_ was added into the mixture and stirred for 1 h. The mixture was diluted with 100 mL deionized water followed with filtration, washing and desiccation to prepare EG. Then, EG was re-dispersed in GO aqueous solution (1 or 2 mg mL^−1^) and ultrasonicated for 30 min, the amount of EG was 3, 5, 7, 9 or 11 mg mL^−1^. A certain amount of suspension was further filtrated, dried and molding press (Ambient temperature, 20 MPa) to fabricate compacted GO/EG films (diameter of 40 mm).

### Preparation of Electrospun PI Fibers

Equimolar ODA and PMDA were added into DMAc successively, and stirred for 30 min at ice bath to get PAA solution. PAA fibers were obtained with the electrospinning technology as follows: collection distance of 30 cm, voltage of 25 kV, injection speed of 0.05 mm min^−1^. PAA fibers were dried for 12 h at 80 °C and then thermal imidized (120 °C/1 h + 200 °C/1 h + 250 °C/1 h, heating rate of 1 °C min^−1^) to get PI fibers.

### Preparation of PI Composite Films

A certain amount of ODA and Fe_3_O_4_ was dispersed in DMAc; the PMDA with equal molar of ODA was added to the above solution and stirred for 30 min at ice bath to get Fe_3_O_4_/PAA mixture (the mass fraction of Fe_3_O_4_ in Fe_3_O_4_/PAA was 0%, 10%, 20%, 30% or 40%). Fe_3_O_4_/PAA was poured on the GO/EG films and spread out by the blade coating, and then put PI fibers on the Fe_3_O_4_/PAA. The whole composites were dried for 12 h at 80 °C and then imidized with the same procedure of PI fibers to get PI composite films. The components of PI composite films are shown in Table S1.

### Characterization

Morphologies of PI composite films were observed by scanning electron microscope (SEM, Verios G4, FEI Corporation, USA). The *λ* of PI composite films was measured by the transient plane heat source method (TPS2200, Hot Disk, Sweden) according to ISO 22007-2. Slab and thin film module were chosen to calculate the in-plane and through-plane *λ* of PI composite films. The surface temperature of PI composite films was recorded by infrared thermography (Ti 300, Fluke, USA). Conductivity tester (RTS-8, Guangzhou Four Probes Technology, China) and Physical Property Measurement System (CFMS-14T, Cryogenic, UK) were utilized to measure the electrical conductivity and magnetic properties. EMI shielding parameters of PI composite films were tested by VNA (MS4644A, Anritsu, Japan) at X-band (8.2–12.4 GHz) according to ASTM D5568-08. The mechanical properties of PI composite films were tested by electronic universal testing machine (CMT 7204, Suns, China) in accordance with ASTM D882-2018. The tensile rate was 2 mm min^−1^, and each sample was measured at least 5 times; the average value was taken.

## Results and Discussions

**Fig. 1:**
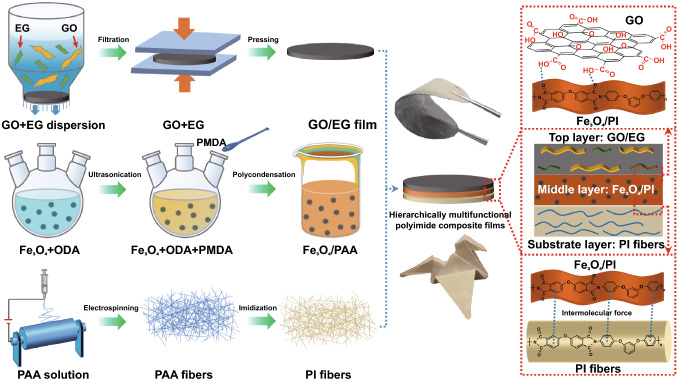
Schematic diagram of the preparation process for PI composite films

Schematic diagram of the preparation process for PI composite films is shown in Fig. 1. The top layer of PI composite films is GO/EG. EG sheet is thicker than GO (Fig. S1), maintaining high C/O atomic ratio (Fig. S3a) and complete crystal structure (Fig. S3b), giving the GO/EG films excellent thermal conductivity and EMI shielding performances [36]. GO sheet seems like flexible cloth (Fig. S1b), which gives the GO/EG film formability and flexibility [37]. As GO has poor thermal conductivity [24], the amount of GO in GO/EG should be as low as possible to make GO/EG obtain high thermal conductivity. In this work, a certain amount of EG was added into the 1 or 2 mg mL^−1^ GO aqueous solutions to judge the formability of GO/EG films. Results show that even a little EG (three times the mass of GO) is added into the 1 mg mL^−1^ GO solution, the GO/EG films are difficult to completely strip off from the filter membrane (Fig. S1c). When the EG whose mass is 5.5 times that of GO is added into the 2 mg mL^−1^ GO solution, the GO/EG films are brittle (Fig. S1e). Therefore, the optimized mass ratio of GO and EG is 2:9 and the GO aqueous solution is 2 mg mL^−1^, the fabricated GO/EG films could be completely peeled off from the filter membrane (Fig. S1d). In addition, the formability of GO/EG films is also related to the volume of GO/EG dispersion during filtration. In this work, the filter membrane cannot be covered if the volume of GO/EG dispersion is less than 3 mL. However, when the volume of GO/EG dispersion exceeds 9 mL, the GO/EG films are too thick to be completely peeled off. The thickness of GO/EG films prepared by 3, 5, 7 and 9 mL of GO/EG dispersion is approximately 20, 35, 50, and 70 μm (Fig. S4a–d, Table S1). The thickness of the PI fibers mat is about 160 μm (Fig. S4e). As can be seen from Fig. 1, PI composite films have excellent flexibility and can be bent or folded into the shape of a paper crane without EG falling off, and there is no separation between layers. The main reason is that GO has good flexibility and high specific surface area to fix EG. The combination of the GO/EG layer and the Fe_3_O_4_/PI layer mainly relies on hydrogen bonding and *π*–*π* interaction [38, 39]. The combination of the Fe_3_O_4_/PI layer and the PI fibers layer relies on the entanglement of PI molecular chains.

**Fig. 2:**
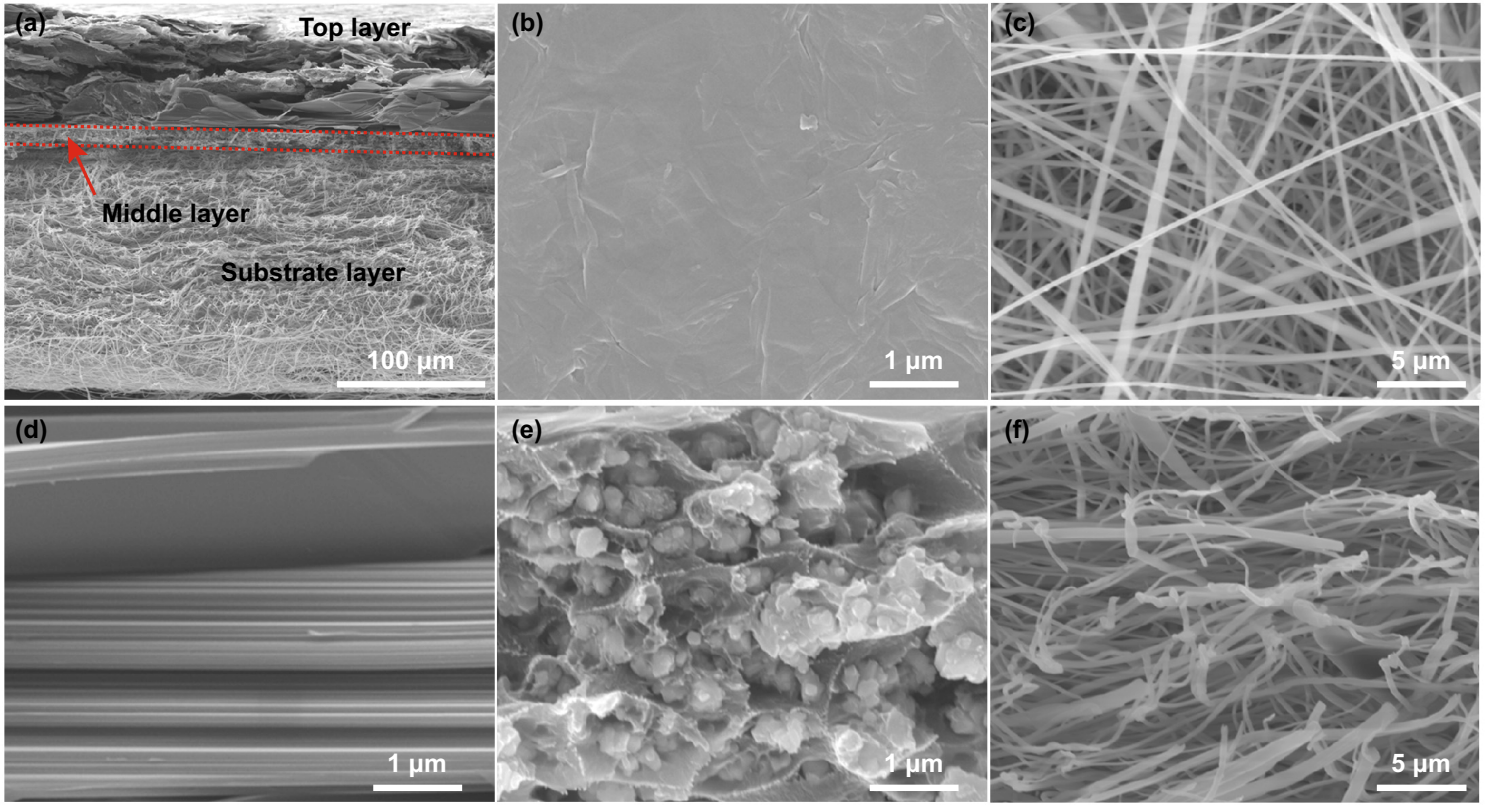
SEM images of PI composite films. Side view (**a**), top view (**b**), bottom view (**c**), magnification of cross section of PI composite films (GO/EG (**d**), Fe_3_O_4_/PI (**e**), PI fibers (**f**))

Figure 2 shows the morphologies of PI composite films. The overall cross section is shown in Fig. 2a. PI composite films have obvious hierarchical structure, and the overall thickness is about 245 μm. The layers of PI composite films are tightly connected to each other. The top layer of PI composite films is GO/EG film with thickness of about 70 μm, and the EG sheets arrange regularly and overlap each other (Fig. 2a, d). The main reason is that the addition of GO greatly improves the dispersibility of EG in water, and the directional flow of water presents an orderly structure during filtration. GO/EG films are compact, and the surface is smooth and has no defects such as wrinkles (Fig. 2b). However, the GO/EG films without molding press have many fragments and gaps, and its surface is uneven (Fig. S2). The middle layer of PI composite films is Fe_3_O_4_/PI with thickness of about 16 μm. Fe_3_O_4_ is completely separated and coated by PI, and PI has an obvious orientation in the horizontal direction (Fig. 2e). The main reason is that the molecular chains of PAA spread in the horizontal direction due to the shearing force in the blade coating process, and the orientation characteristics of the molecular chains are fixed after solvent volatilization and thermal imidization [40]. The substrate layer of PI composite films is PI fibers with thickness of about 160 μm. PI fibers are fluffy and smooth (Fig. 2c, f).

**Fig. 3:**
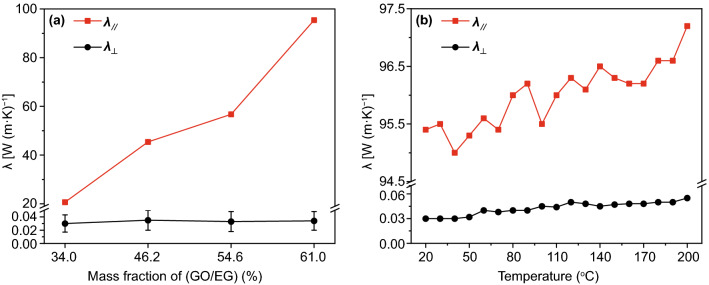
*λ* of PI composite films with fixed addition of Fe_3_O_4_/PI and PI fibers (**a**), *λ* of PI composite films contains 61.0 wt% of GO/EG change with temperature (**b**)

By comparing the thermal conductivity coefficient of PI composite films with different components (Fig. S5), it can be found that the through-plane thermal conductivity coefficient (*λ*_⊥_) of PI composite films has no relationship with the amount of each layer, and remains at about 0.03 W (m K)^−1^. The in-plane thermal conductivity coefficient (*λ*_∥_) of PI composite films is directly related to the amount of GO/EG, and is hardly affected by the amount of Fe_3_O_4_/PI and PI fibers. The *λ*_∥_ of PI composite films with fixed addition of Fe_3_O_4_/PI and PI fibers (39 and 25 mg, respectively) reaches maximum of 95.40 W (m K)^−1^ when the amount of GO/EG is 61.0 wt% (Fig. 3a). The main reason is that the thickness of PI fibers layer of PI composite films is much thicker than that of the GO/EG layer and the Fe_3_O_4_/PI layer. The heat flow in the direction perpendicular to the plane of PI composite films will lose lots of energy when it is conducted through the PI fibers with low thermal conductivity, causing low *λ*_⊥_ of PI composite films. When the amount of PI fibers in PI composite films is increased (that is, increasing the thickness of PI fibers mat), the conduction process of heat flow in the direction perpendicular to the PI composite films plane hardly changes, causing the *λ*_⊥_ of PI composite films basically unchanged. In the direction of the PI composite films plane, GO/EG constructs perfect thermal conduction pathways and network, so that the heat flow can be quickly conducted under lower thermal resistance, giving PI composite films high *λ*_∥_. The more GO/EG, the more perfect of the thermal conduction pathways and network formed, which is conductive to the further enhancement in the *λ*_∥_ of PI composite films. Based on the above results, this work fixes the thickness of GO/EG and PI fibrous layer of 70 μm (~ 100 mg) and 160 μm (~ 25 mg) to make PI composite films possess the best thermal conductivity and thin thickness. The thermal conductivity of PI composite films varies with temperature as shown in Fig. 3b. It can be seen that both *λ*_∥_ and *λ*_⊥_ of PI composite films increase slightly as the temperature increases. This is because the thermal motion of the molecules increases with the temperature increases [41]. The thermal conductivity stability of PI composite films is evaluated by testing its thermal conductivity coefficient after bending (Fig. S6). The thermal conductivity ratio of PI composite films before and after bending is stable at ~ 1, indicating that its thermal conductivity does not change much after bending, showing good *λ* stability.

**Fig. 4:**
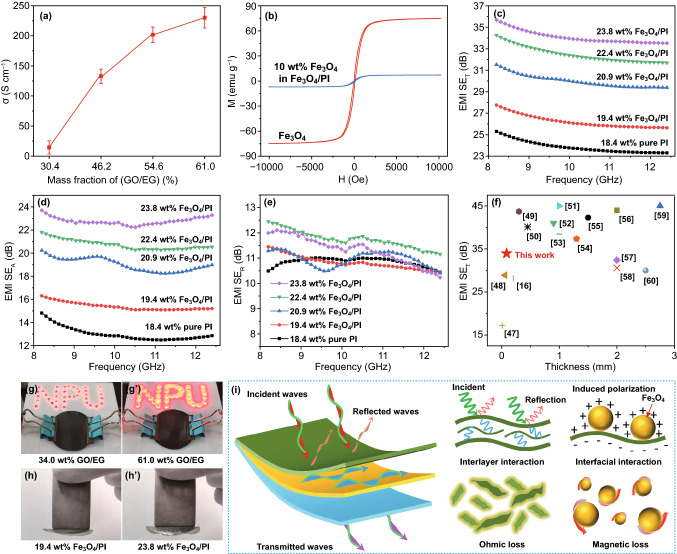
Surface electrical conductivity of PI composite films with fixed addition of Fe_3_O_4_/PI and PI fibers (**a**), magnetization curves of Fe_3_O_4_/PI and Fe_3_O_4_ (**b**), EMI SE_T_ (**c**), EMI SE_A_ (**d**) and EMI SE_R_ (**e**) of PI composite films, summarization of EMI SE of PI composite films and other works (**f**), the digital photos showing electrical conductivity (**g**, **g′**) and magnetic properties (**h**, **h′**) of PI composite films, EMI shielding mechanism of PI composite films (**i**)

Surface electrical conductivity (*σ*) of PI composite films increases with the amount of GO/EG increases (Fig. 4a). The *σ* of PI composite films with fixed addition of Fe_3_O_4_/PI and PI fibers (39 and 25 mg, respectively) reaches the maximum of 230.0 S cm^−1^ when the amount of GO/EG is 61.0 wt%. Connecting the PI composite films with the least and the largest amount of GO/EG into the light-emitting diode (LED) circuit (Voltage is 10 V, LED lights are connected in parallel), the LED brightness of the latter is brighter (Fig. 4g, g′), indicating lower resistance. However, the *σ* of PI composite films has no relationship with the amount of Fe_3_O_4_/PI (Fig. S7), ascribing Fe_3_O_4_/PI located in the middle of PI composite films. The saturation magnetization of pure Fe_3_O_4_ and Fe_3_O_4_/PI (10 wt.%) is 74.8 and 7.1 emu g^−1^, respectively (Fig. 4b). PI composite films can be easily attracted by the magnet (Fig. 4h, h′). Under the condition of fixed amount of the top and substrate layer of PI composite films (GO/EG: 100 mg, PI fibers: 25 mg), the mass fraction of Fe_3_O_4_/PI in PI composite films increases with the proportion of Fe_3_O_4_ in Fe_3_O_4_/PI increases. From Fig. 4c, d, both the total EMI SE (EMI SE_T_) and the absorption efficiency (EMI SE_A_) of the PI composite films increase with the percentage of Fe_3_O_4_/PI increases. When the middle layer of PI composite films is pure PI, the EMI SE_T_ and EMI SE_A_ are the lowest, about 23.8 and 13.0 dB, respectively, which increase to 34.0 and 22.8 dB when the middle layer is Fe_3_O_4_/PI and its amount is 23.8 wt%. However, the reflection efficiency (EMI SE_R_) of PI composite films is less affected by the amount of Fe_3_O_4_/PI, and is basically maintained at about 11.0 dB (Fig. 4e). The main reason is that the EMI SE_R_ is mainly related to the electrical conductivity [42]. In the case of fixed amount of GO/EG, the electrical conductivity of the PI composite films is basically unchanged, and the EMI SE_R_ has little change. According to the structural characteristics of PI composite films, its EMI shielding mechanism (Fig. 4i) may be concluded as follows. First, the incident electromagnetic wave excites high-frequency oscillating current on the surface of the GO/EG due to the impedance mismatch between GO/EG and the ambient space, reflecting part of the electromagnetic wave into the atmosphere. The remaining electromagnetic wave transmits to the inside of the GO/EG, and is reflected and scattered multiple times between the EG sheets [43]. In this process, each wavelet could interfere with each other and attenuate gradually. In addition, the highly oriented EG provides a large number of dipoles, which increases the loss ability of electromagnetic wave. After the electromagnetic wave enters the Fe_3_O_4_/PI, the interface polarization is induced under the action of the external field, resulting in polarization loss due to the different electrical conductivity of the GO/EG and the Fe_3_O_4_/PI. In addition, the Fe_3_O_4_ further loses electromagnetic wave in the form of natural resonance [44–46]. The EMI SE of PI composite films and other materials based on graphite derivatives and magnetic substances is summarized in Fig. 4f [16, 47–60]. It can be seen that the PI composite films prepared in this work has a relatively high specific efficiency (the ratio of EMI SE to the effective thickness of the material, and the effective thickness of PI composite films for EMI shielding is about 85 μm), which is about 400 dB mm^−1^.

**Fig. 5:**
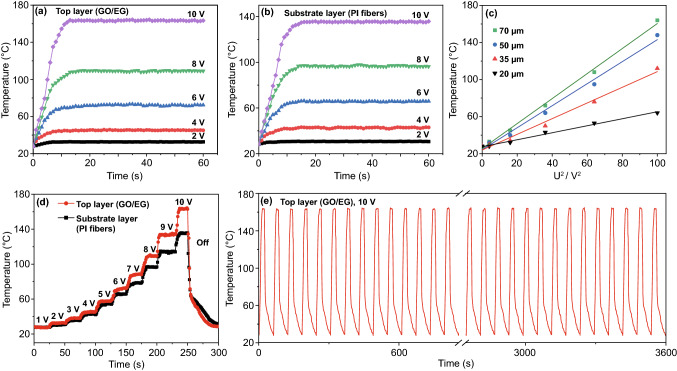
Electric-heating property of PI composite films. The time–temperature relationship of PI composite films with different voltages (**a**, **b**), linear fitting of experimental temperature versus *U*^2^ (**c**), surface temperatures of the PI composite films upon gradiently changed voltages (**d**), electric-heating stability of the PI composite films upon repeated supplied voltages (**e**)

PI composite films shows good electric-heating properties based on the Joule effect, which could be used in the fields including defogging and defrosting. Fast electric-heating response and excellent thermal conductivity can ensure rapid generation and conduction of a large amount of heat, which can reduce energy waste. The electric-heating results of PI composite films (GO/EG: 61.0 wt%, Fe_3_O_4_/PI: 23.8 wt%, PI fibers: 15.2 wt%) with the best thermal conductivity and EMI shielding performance are shown in Fig. 5. Figure 5a, b shows the time–temperature relationship of PI composite films with different voltages. The temperature of the top and substrate layer at stable condition increases with the voltage increases, indicating that more Joule heat is generated. The temperature of the substrate layer of PI composite films is lower than the top layer in the condition of the same voltage. For example, the temperature of the top layer of the PI composite films with voltage of 10 V is about 163 °C, while substrate layer is about 134 °C. The main reason is that the top layer of PI composite films is conductive, which generates heat due to the Joule effect. The substrate layer of PI composite films is insulated, which cannot generate Joule heat. The heat of the top layer of PI composite films is partially lost in the process of conducting to the substrate layer, and the low through-plane thermal conductivity of PI composite films hinders the heat diffusion through the layers, resulting in the lower temperature of substrate layer. In order to fully understand the electric-heating mechanism, thermodynamic analysis is conducted. Based on the principle of energy conservation, the consumed electrical energy is equal to the generated thermal energy when the temperature of PI composite films is stable. The surface temperature of PI composite films follows Eq. 1 [61]:

1 $$T_{{\text{s}}} = T_{0} + \frac{{U^{2} }}{RhA}$$

where *T*_0_ and *T*_s_ are the initial and stable temperature of the PI composite films, *U* is the voltage applied to the PI composite films, *h* is the convective heat transfer coefficient, and *R* and *A* are the resistance and surface area of the PI composite films, respectively.

The relationship between the temperature of the top layer of the PI composite films and the square of the applied voltage is shown in Fig. 5c. It can be seen that *T*_s_ has a linear relationship with *U*^2^, and the slope is 1/*R*. This indicates that the thicker the GO/EG, the higher perfection of the conductive networks and the lower the resistance.

Figure 5d shows the relationship between the surface temperature of the PI composite films and the gradient voltage. Surface temperature of the PI composite films stabilized quickly when the voltage gradually increases with the gradient of 1 V, and the response time is about 5 s. The temperature of the top layer of the PI composite films is always higher than the substrate layer. The higher the voltage, the greater the temperature differences. When the voltage is cut off, the surface temperature of the PI composite films decreases rapidly, and the cooling rate slows down after 5 s. To evaluate the reliability and stability of the electric-heating property, periodic constant voltage is applied to the PI composite films (The voltage is 10 V. Each cycle lasts 60 s, the voltage is applied for the first 20 s, and then cut off), the result is shown in Fig. 5e. The temperature of the top layer of PI composite films is stable at about 163 °C. At the beginning of each cycle, the temperature rises rapidly to be stable, and then the temperature drops rapidly to about 60 °C and then slowly decreases when the voltage is cut off. The process lasts for 6 h, and the temperature changes of PI composite films has good repeatability.

**Fig. 6:**
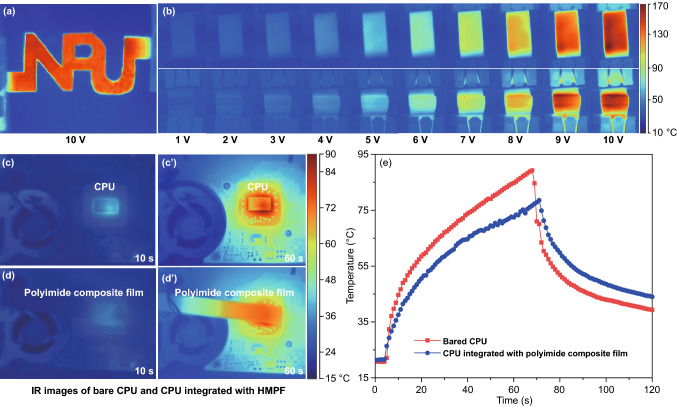
Infrared thermal images of PI composite films. IR images of PI composite films with the shape of “NPU” at 10 V voltage (**a**), IR images of the PI composite films upon bending and stretching (**b**), IR images of bare CPU (**c**, **c′**) and integrated with PI composite films (**d**, **d′**), the working temperature of CPU (**e**)

Infrared thermal (IR) images can clearly reflect the thermal performance of PI composite films. Figure 6a shows the electric-heating performance (applied voltage of 10 V) of the PI composite films with a shape of “NPU.” The temperature distribution in the PI composite films is uniform, indicating the PI composite films has uniform thermal/electrical conduction networks. Figure 6b shows the IR images of the PI composite films upon bending and stretching. The uniform temperature distribution during the bending indicates the bending has not destroyed the thermal/electrical conduction networks. The actual heat dissipation of PI composite films is characterized by the temperature change of the central processing unit (CPU) of computer. The temperature of the bared CPU (Fig. S8a) rises rapidly during working (Fig. 6c, c′), the surface temperature rises to 90 °C in 70 s, and slowly drops to 40 °C after the power is turned off (Fig. 6e). The surface temperature of the CPU integrated with PI composite films (Fig. S8b) rises slowly (Fig. 6d, d′), which is 78 °C at 70 s (12 °C lower than that of the bare CPU), and it drops slowly after turning off the power. It shows that the PI composite films can effectively reduce the working temperature of CPU, and preventing the temperature of CPU from changing sharply.

**Fig. 7:**
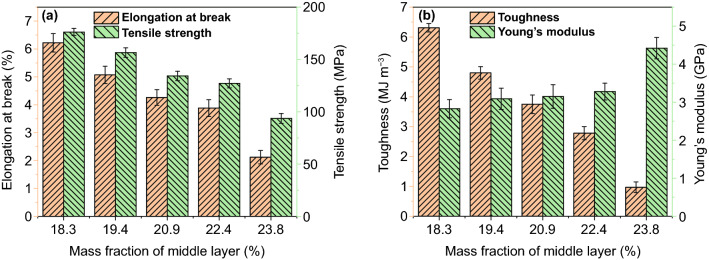
Tensile strength and elongation at break (**a**), toughness and Young's modulus (**b**) of PI composite film

Figure 7 shows the tensile strength, elongation at break, toughness and Young's modulus obtained from the stress–strain curve of PI composite films (Fig. S9). Tensile strength, elongation at break and toughness of PI composite films decrease with the increase in the amount of Fe_3_O_4_/PI, while the Young's modulus increases when the amount of Fe_3_O_4_/PI increases. When Fe_3_O_4_/PI amount is 23.8 wt%, the tensile strength, elongation at break, toughness and Young's modulus of PI composite films are about 93.6 MPa, 2.12%, 0.97 MJ m^−3^ and 4.42 GPa, respectively. The increase in the amount of Fe_3_O_4_/PI in PI composite films is mainly due to the increase in the amount of Fe_3_O_4_ in Fe_3_O_4_/PI, which increases the defects of PI [62].

## Conclusion

In this work, the obtained hierarchically multifunctional PI composite films have a three-layer structure, the top layer is GO/EG for thermal conduction and EMI shielding, the middle layer is Fe_3_O_4_/PI for enhancing the EMI shielding effectiveness, the substrate layer is PI fibers for improving the mechanical property. The top, middle and substrate layers of PI composite films are tightly bonded. PI composite films with 61.0 wt% and 23.8 wt% of GO/EG and Fe_3_O_4_/PI, respectively, possess the highest *λ*_∥_ (95.40 W (m K)^−1^), EMI SE (34.0 dB), excellent tensile strength (93.6 MPa) and fast electric-heating response (5 s, voltage gradient is 1 V). Taking the CPU as the actual heat dissipation scenario verifies that PI composite films has broad application prospects in the field of light and miniaturized electronic equipment.

## Supplementary Information

Below is the link to the electronic supplementary material.

Supplementary file1 (PDF 927 kb)
