# Supplementary material for: Hierarchically Multifunctional Polyimide Composite Films with Strongly Enhanced Thermal Conductivity
Source: Nanomicro Lett. 2021 Dec 10;14:26. doi: 10.1007/s40820-021-00767-4 (PMC8664909; doi:10.1007/s40820-021-00767-4)
Supplement: Supplementary file 1 — Supplementary file1 (PDF 927 kb) [file 40820_2021_767_MOESM1_ESM.pdf]

Supporting Information for

Hierarchically Multifunctional Polyimide Composite Films with Strongly Enhanced Thermal Conductivity

Yongqiang Guo[1], Hua Qiu[1, *], Kunpeng Ruan[1], Yali Zhang[1], Junwei Gu[1, *]

MOE Key Laboratory of Material Physics and Chemistry under Extraordinary Conditions, Shaanxi Key Laboratory of Macromolecular Science and Technology,School of Chemistry and Chemical Engineering, Northwestern Polytechnical University, Xi'an, Shaanxi, 710072, P. R. China

Corresponding authors. E-mail: huaqiu@nwpu.edu.cn (H. Qiu), gjw@nwpu.edu.cn & nwpugjw@163.com (J. Gu)

Supplementary Figures and Table

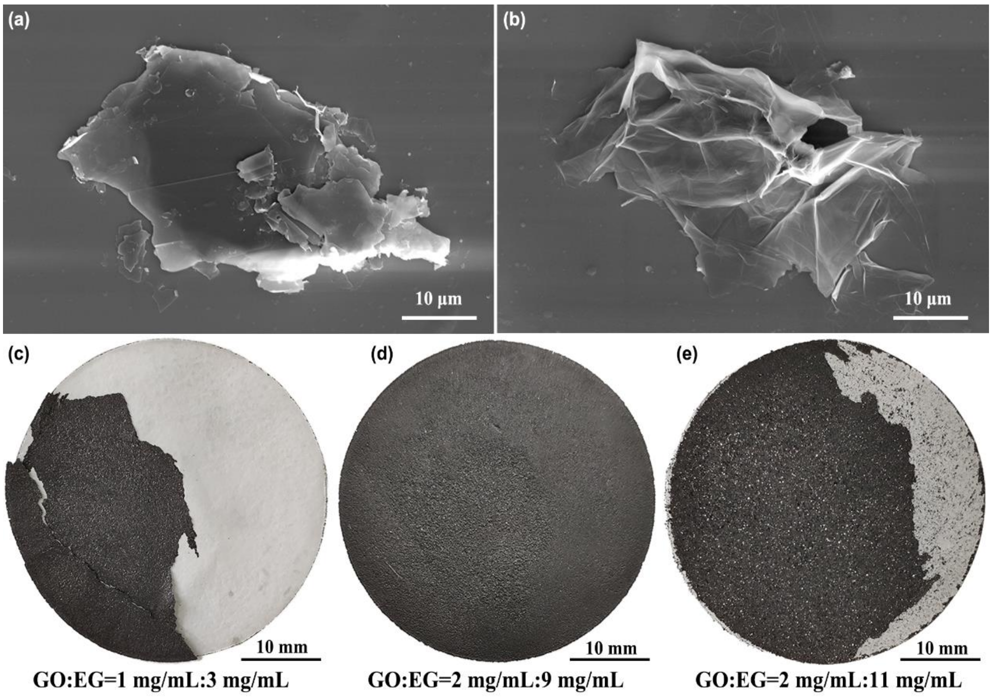

Fig. S1 SEM images of EG (**a**) and GO (**b**), photos of GO/EG films with different mass ratio of GO and EG (**c, d**)

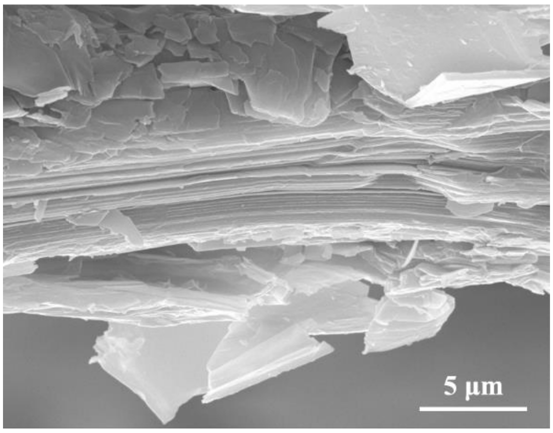

Fig. S2 SEM image of cross-section for uncompacted GO/EG films

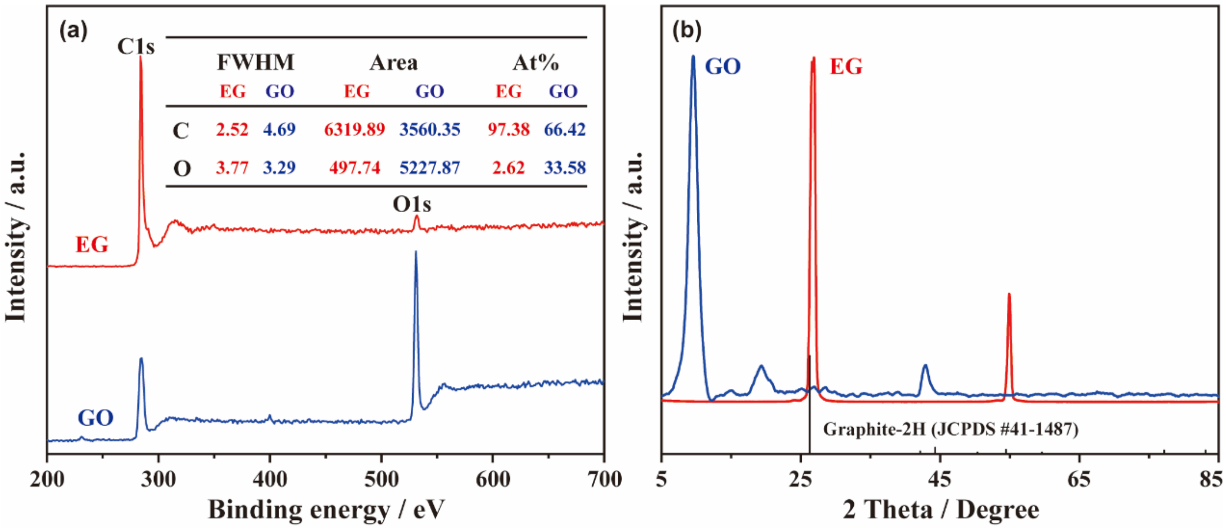

Fig. S3 Characterization of EG and GO. XPS (**a**), XRD (**b**)

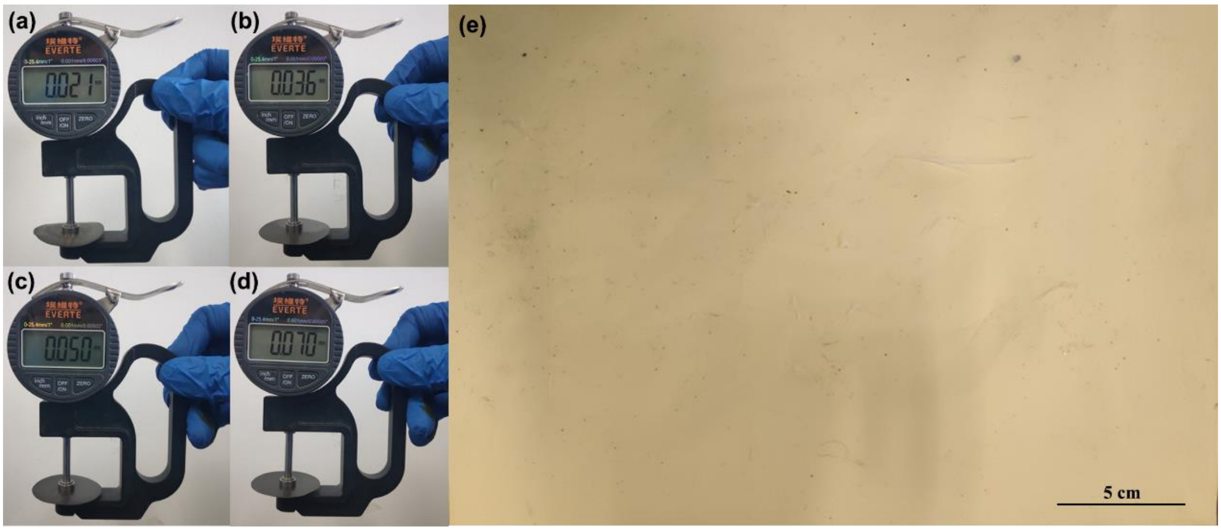

Fig. S4 Photos of GO/EG films with different thickness (**a-d**) and PI fibers (**e**)

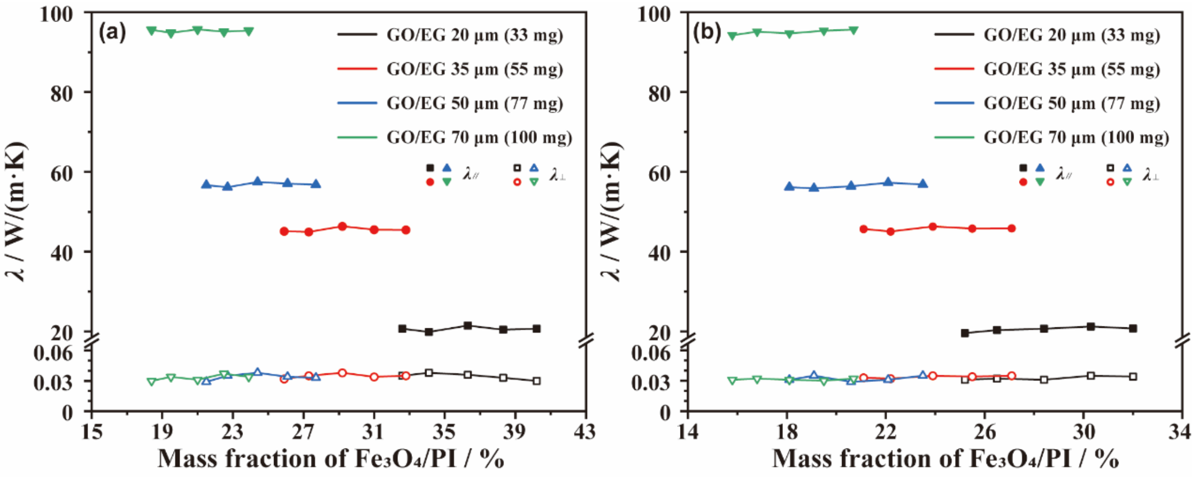

Fig. S5 Influence of GO/EG, Fe_3O_4/PI and PI fibers on the thermal conductivity of PI composite films. The mass of PI fibers in PI composite films is about 25 mg (**a**) and 50 mg (**b**)

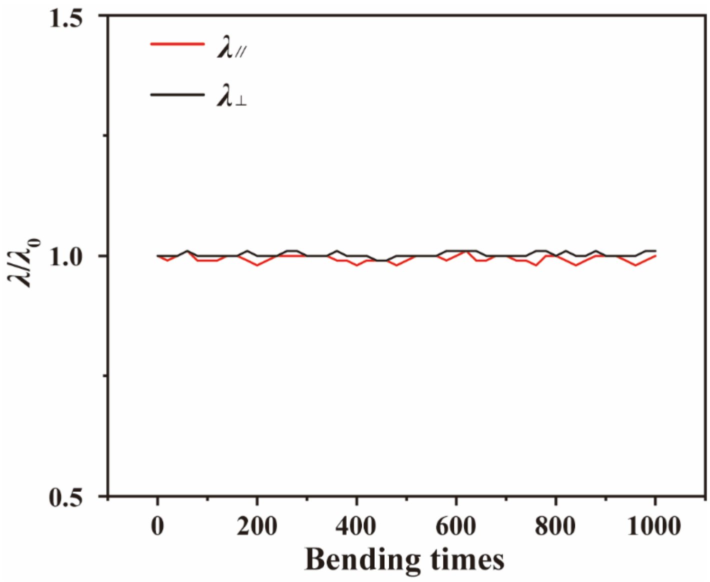

Fig. S6 Relative thermal conductivity coefficients of PI composite films upon repeated bending and stretching

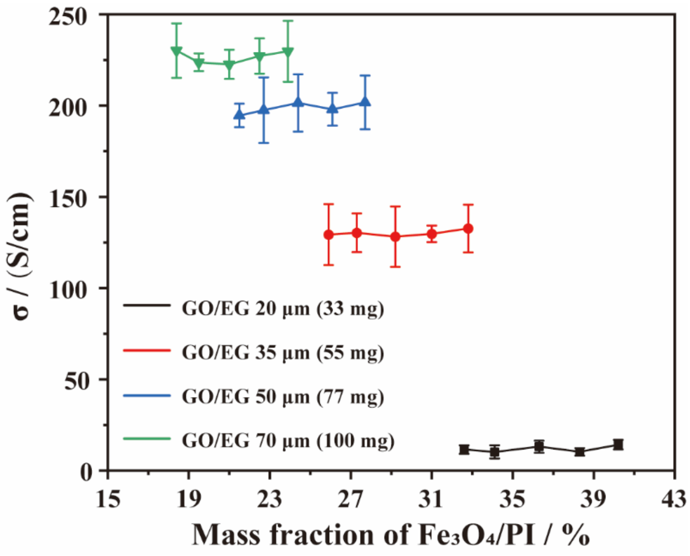

Fig. S7 Influence of GO/EG and Fe_3O_4/PI on the surface electrical conductivity of PI composite films with 25 mg of PI fibers

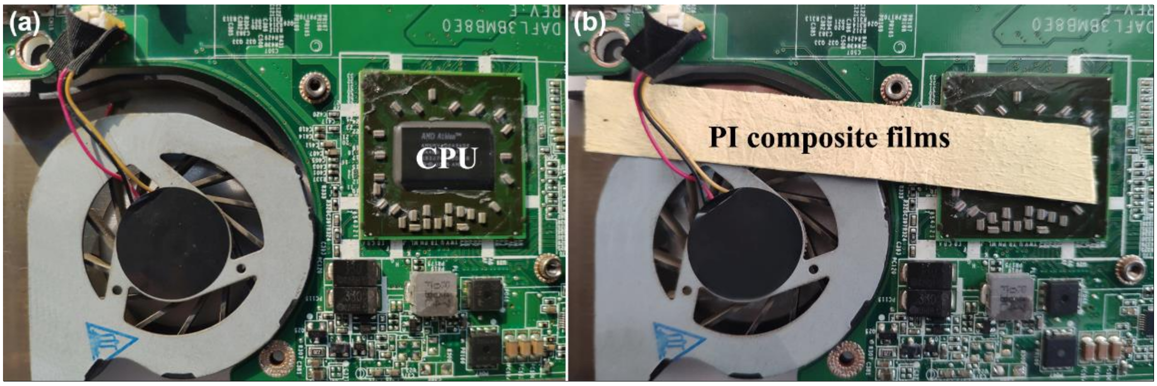

Fig. S8 Photos of bared CPU (**a**) and CPU integrated with PI composite films (**b**)

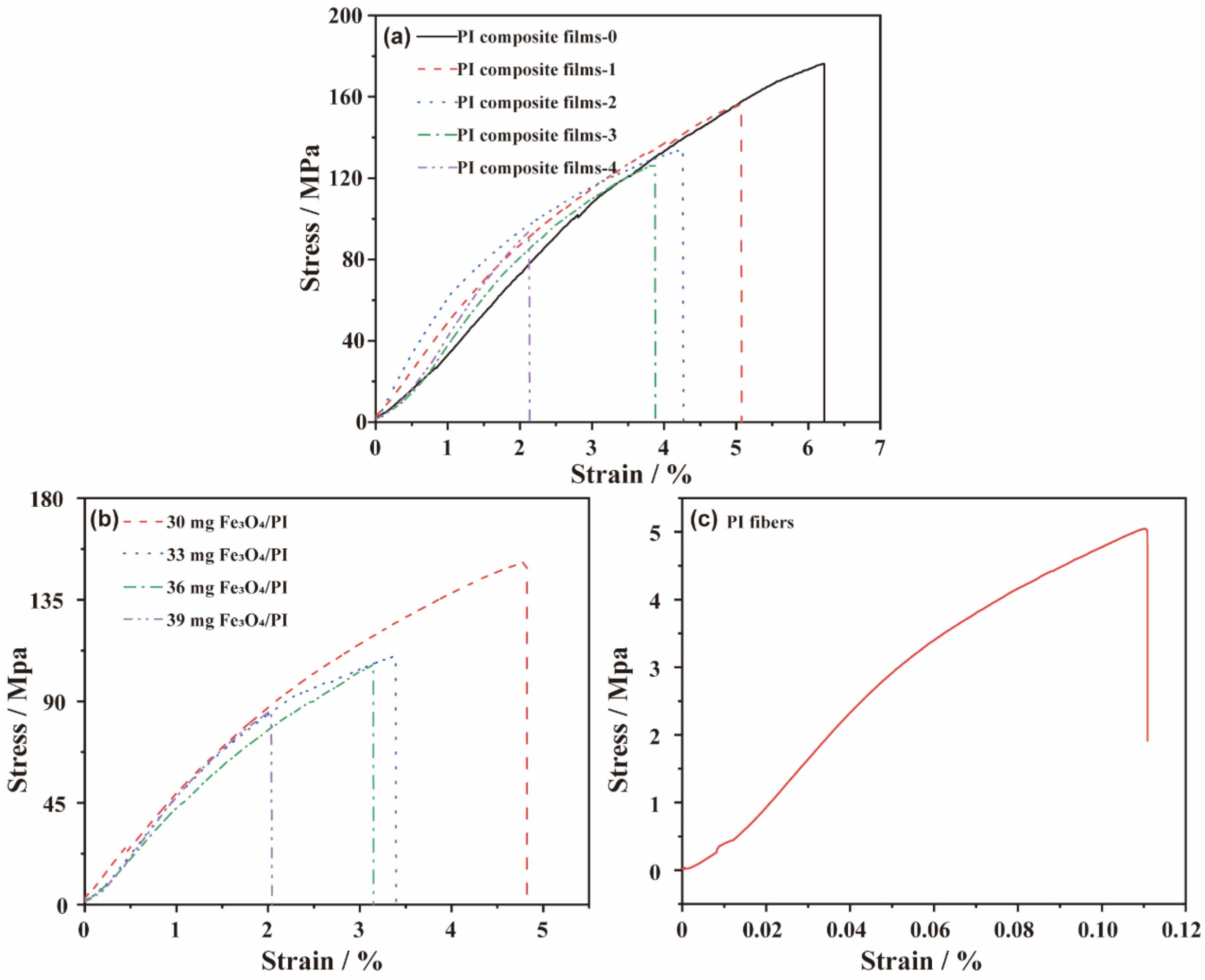

Fig. S9 The stress-strain curves of PI composite films (**a**), Fe_3O_4/PI films (**b**) and PI fibers mat (**c**)

Table S1 The amount of component in PI composite films

Top layer: GO/EG		Middle layer: Fe_3O_4/PI*			Substrate layer: PI fibers	
Thickness (μm)	Mass (mg)	Thickness (μm)	Mass (mg)	Mass fraction of Fe_3O_4 (%)	Thickness (μm)	Mass (mg)
20±2	33±3		28±3	0	160±10	25±2
35±2	55±3		30±4	10	320±10	50±2
50±2	77±3	16±2	33±4	20	--	--
70±2	100±3		36±4	30	--	--
--	--		39±5	40	--	--

*Label PI composite films with 28 mg, 30 mg, 33 mg, 36 mg and 39 mg of Fe_3O_4/PI as PI composite films-x (x=0, 1, 2, 3, 4), respectively.
